# Comparative Anatomy of the Teeth

**Published:** 1852-01

**Authors:** 


*Comparative Anatomy of the Teeth.*—With the present number of the Journal, we commence the republication from the Encyclopedia of Anatomy and Physiology, the article by Professor Owen, on the Comparative Anatomy of the Teeth. The wood engravings with which it is illustrated, made by Mr. Johnson of Baltimore, will, for neatness of execution, we think, compare with the original. The ability and profound research exhibited in the preparation of the article by the distinguished author, will secure for it from the more scientific of our readers, we feel well assured, an attentive perusal.

